# Necrotizing Pneumonia in a Vaccinated Child: A Rare Complication of Viral and Bacterial Co-infection

**DOI:** 10.7759/cureus.99467

**Published:** 2025-12-17

**Authors:** Pradeep Kumar Elangovan, Sangeetha Hariprasath

**Affiliations:** 1 Pediatrics, Gheeth IVF Hospital, Kanyakumari, IND; 2 Pediatrics, Apollo Hospitals, Chennai, IND

**Keywords:** empyema thoracis, necrotizing pneumonia, pediatric thoracoscopy, pleural effusion, pneumococcal infection

## Abstract

Necrotizing pneumonia is a rare but serious complication of community-acquired pneumonia in children, often associated with parenchymal destruction, cavitation, and empyema. Despite the availability of pneumococcal conjugate vaccines, necrotizing pneumonia may still occur in immunized and otherwise healthy children due to non-vaccine serotypes or viral-bacterial co-infections.

We report the case of a three-year-old developmentally normal female child who had received all age-appropriate immunizations, including three doses of the pneumococcal polysaccharide conjugate vaccine (13-valent, adsorbed; Prevnar), and presented with fever, tachypnea, and chest retractions. Initial evaluation revealed severe anemia, marked leukocytosis, and a significantly elevated C-reactive protein level. Imaging demonstrated bilateral lower lobe consolidation with cavitations and a right-sided loculated pleural effusion. Respiratory viral panel testing was positive for influenza A, parainfluenza, and rhinovirus, while pneumococcal antigen was detected in pleural fluid. Blood and pleural fluid cultures were negative, and pleural fluid analysis was consistent with an exudate showing neutrophilic predominance.

The child received broad-spectrum antimicrobials along with oseltamivir, but persistent fever and respiratory distress necessitated surgical management. On the third day of admission, video-assisted thoracoscopic surgery with decortication was performed, revealing thick pleural peel and pus collections. Following surgery, the child showed progressive clinical improvement with resolution of fever, decreasing inflammatory markers, and expansion of the lung. She was discharged hemodynamically stable on oral medications.

This case highlights the importance of early recognition of necrotizing pneumonia even in vaccinated and immunocompetent children. Viral-bacterial synergy plays a significant role in disease progression, and negative cultures should not delay diagnosis when supported by clinical, radiological, and antigen test findings. Prompt multidisciplinary care and timely surgical intervention remain crucial for favorable outcomes.

## Introduction

Necrotizing pneumonia (NP) is a rare but serious complication of community-acquired pneumonia in children, with a prevalence of approximately 0.01% among pediatric hospitalizations, often associated with parenchymal destruction, cavitation, and empyema [1]. First recognized in the late 1990s as a distinct clinicopathological entity, NP represents one of the most destructive forms of bacterial pneumonia in the pediatric population [2].

Over the past two decades, the incidence of NP has increased worldwide, partly due to the emergence of antibiotic-resistant pathogens and infections with non-vaccine serotypes of Streptococcus pneumoniae and Staphylococcus aureus [3]. Although the introduction of pneumococcal conjugate vaccines (PCV) has markedly reduced the global burden of invasive pneumococcal disease, cases of NP continue to occur, even among immunized and otherwise healthy children [4].

Respiratory viruses, particularly influenza, parainfluenza, and rhinovirus, predispose children to secondary bacterial infection by injuring the airway epithelium and dysregulating innate immunity, which enhances bacterial adhesion and invasion [5]. In influenza, epithelial activation drives neutrophil recruitment; superinfection with Panton-Valentine leukocidin (PVL)-producing Staphylococcus aureus then triggers rapid neutrophil lysis and protease release, causing epithelial disintegration and extensive parenchymal necrosis [5,6]. This synergistic viral-bacterial interaction contributes to the development of severe necrotizing forms of pneumonia even in previously healthy children.

Early recognition remains challenging because cultures are frequently negative, especially when empirical antibiotics are initiated before microbiological sampling [1,2]. Ultrasound of the chest is recommended as the initial imaging modality in children with complicated pneumonia, as it can accurately detect pleural effusion, septations, and early parenchymal changes without radiation exposure [7]. Computed tomography (CT) serves as a complementary tool, providing detailed visualization of parenchymal necrosis and cavitation when complications are suspected [2,7]. Rapid molecular and antigen detection assays performed on respiratory specimens such as nasopharyngeal or oropharyngeal swabs, endotracheal aspirates, bronchoalveolar lavage fluid, or pleural fluid aid in identifying the causative organisms.

Management requires a multidisciplinary approach. In the acute phase, conservative management with antibiotics and supportive care remains the treatment of choice [2]. Pleural drainage or fibrinolytic therapy is considered when there is a significant or loculated effusion, while surgical interventions such as video-assisted thoracoscopic surgery (VATS) or decortication are reserved for cases that fail to improve with medical and less invasive management [4,6]. This stepwise approach helps achieve resolution while minimizing unnecessary operative procedures and preserving long-term lung function [2,4].

We present a rare case of necrotizing pneumonia in a previously healthy, vaccinated three-year-old child, complicated by empyema and requiring surgical decortication. Given these complexities, timely diagnosis and individualized management are essential to improve outcomes in children with necrotizing pneumonia.

## Case presentation

A three-year-old, developmentally normal, female child presented with 10 days of high-grade fever associated with progressive respiratory distress. On the second day of illness, the patient was evaluated by a local practitioner and started on oral cefixime. The initial C-reactive protein (CRP) was 14 mg/L, and the total leukocyte count was within normal limits. There was no travel history or known tuberculosis contact.

On the eighth day, due to worsening symptoms, the child was referred to our centre. At presentation, the child was toxic and tachypneic with the following vitals: temperature 102 °F, heart rate 132/min, respiratory rate 62/min, SpO₂ 88-90% on room air, and blood pressure 96/60 mm Hg. Clinical examination revealed bilateral subcostal and intercostal retractions and coarse crepitations over both lung fields.

The child required oxygen supplementation via nasal prongs, maintaining oxygen saturation above 94%, but did not require mechanical ventilation.

Growth parameters were normal, with no failure to thrive or malnutrition. A well-formed Bacille Calmette-Guérin (BCG) scar was present. There were no ENT, skin, mucosal, or lymphoid abnormalities, and no hepatosplenomegaly. The child also had no history of recurrent respiratory, gastrointestinal, or urinary infections.

Laboratory investigations at presentation are summarized in Table 1.

**Table 1:** Laboratory parameters of the patient at presentation and during hospitalization Serial CRP levels showed a marked reduction from 338 mg/L at admission to 30.7 mg/L during recovery. CRP - C-reactive protein; RBC - red blood cell count; WBC - white blood cell count; MTB - mycobacterium tuberculosis

Parameter	Result	Reference range	Unit
Hemoglobin	6.9	11.0-14.5	g/dL
WBC count	28.5	4.0-12.0	×10⁹/L
Neutrophils	90	40-70	%
CRP	338	<10	mg/L
Pleural fluid appearance	Brown, turbid	-	-
Pleural fluid RBC count	1.0×10⁶	0-5	cells/mm³
Pleural fluid WBC count	102.5×10⁹	<0.5	×10⁹/L
Pleural fluid neutrophils	90	<50	%
Pleural fluid lymphocytes	10	<50	%
Pleural fluid protein	Elevated	<3.0	g/dL
Pleural fluid glucose	Low	>40	mg/dL
Pleural fluid Gram stain	Many pus cells, few gram-positive cocci in pairs	-	-
Gastric juice Xpert^®^ MTB	Not detected	-	-
Streptococcus pneumoniae antigen	Positive	Negative	-
Respiratory viral panel	Influenza A, parainfluenza, rhinovirus - positive	Negative	-
Blood culture	Negative	Negative	-
Pleural fluid culture	Negative	Negative	-

**Figure 1:**
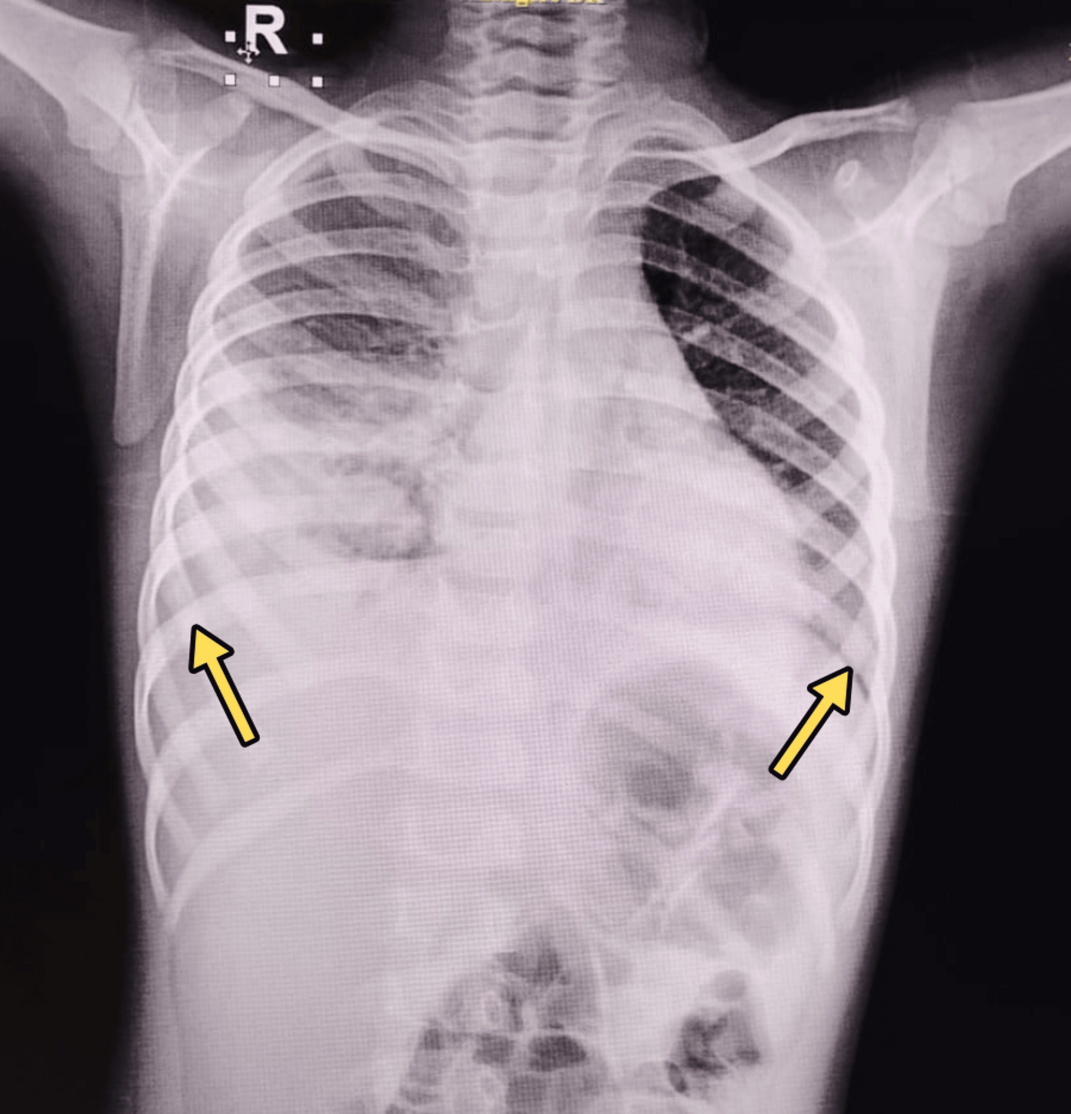
Chest radiograph at admission showed features of pneumonia Chest radiograph demonstrating diffuse consolidation in both lower lobes (arrows).

Chest radiograph at admission showed features of pneumonia with bilateral lower lobe consolidation (Figure 1).

The child was initiated on intravenous ceftriaxone, doxycycline, and linezolid. Oseltamivir was added following the respiratory viral panel results performed on nasopharyngeal swab samples, which were positive for Influenza A, parainfluenza, and rhinovirus.

Point-of-care ultrasound (POCUS) of the chest revealed a right-sided loculated pleural effusion. Given the clinical suspicion of empyema, an ultrasound-guided diagnostic thoracentesis was performed to characterize the fluid. This yielded approximately 10-12 mL of thick, purulent exudate, confirming empyema. The aspiration was limited by the organized nature of the collection, indicating the need for more invasive management.

**Figure 2:**
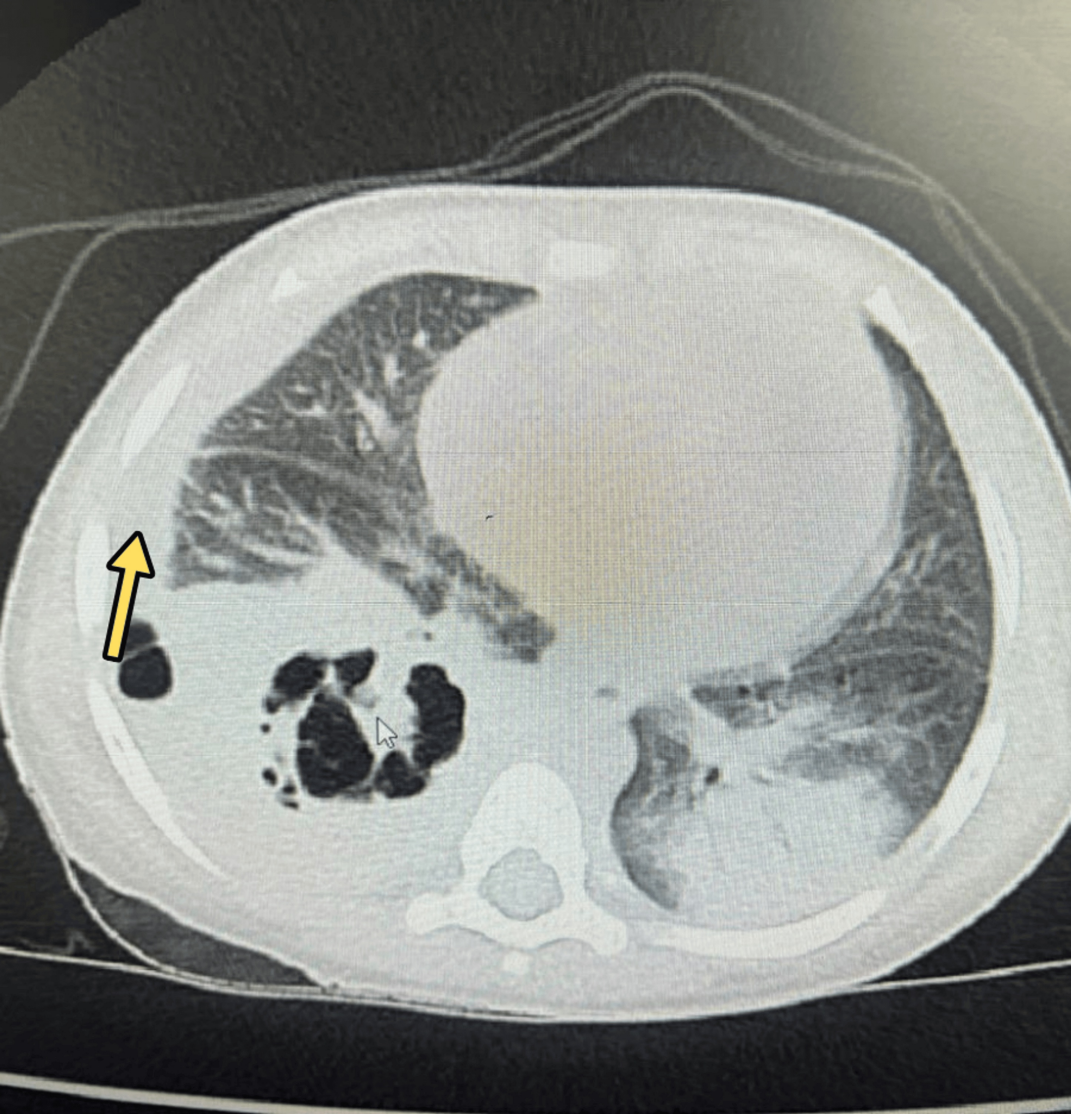
Right pleural effusion Contrast-enhanced computed tomography of the chest showing dense consolidation in both lower lobes with right pleural effusion (yellow arrow).

**Figure 3:**
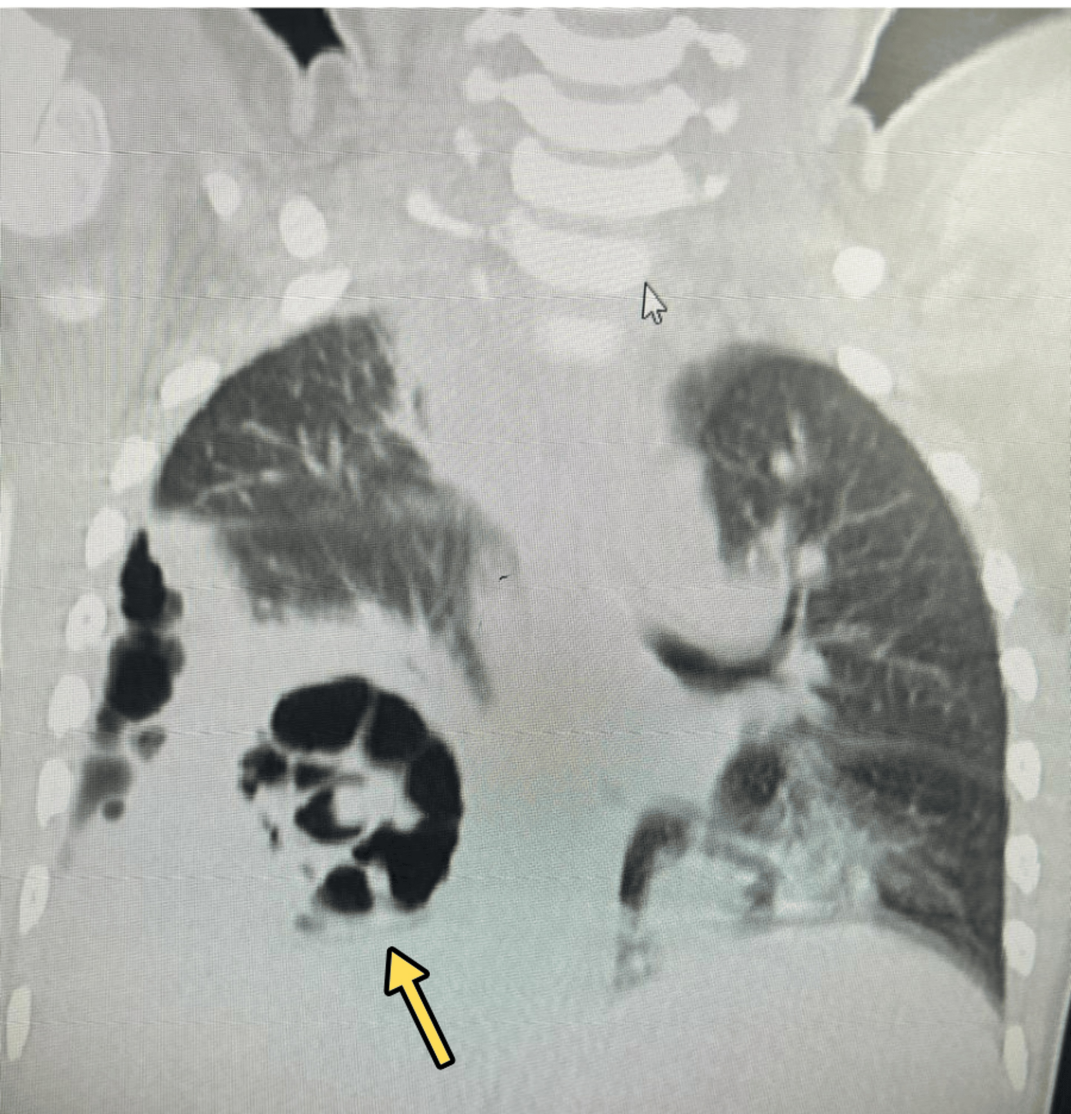
Contrast-enhanced computed tomography (CECT) of the chest showing necrotizing pneumonia Contrast-enhanced computed tomography of the chest with contrast showing dense consolidation in both lower lobes with cavitations (arrow), consistent with necrotizing pneumonia.

Due to persistent fever spikes and tachypnea, a contrast-enhanced CT (CECT) of the chest was obtained, which revealed dense consolidation in both lower lobes with right pleural effusion (Figure 2) and multiple cavitations and necrotizing changes (Figure 3).

A pediatric surgery consultation was sought, and VATS was planned. Packed red blood cell (PRBC) transfusion was administered preoperatively to correct anemia.

On the third day of admission, the child underwent thoracoscopic decortication. Intraoperative findings included a thick pleural peel, multiple loculated pus pockets, and a collapsed right lower lobe, which expanded well after decortication. A right-sided intercostal drain (ICD) was placed after the procedure.

Postoperatively, the child showed steady improvement. The ICD drained approximately 20 mL/day until removal on the sixth postoperative day. CRP and leukocyte counts progressively declined. Pneumococcal antigen was positive in the pleural fluid. In view of intermittent fever, antibiotics were escalated to meropenem and linezolid after intraoperative cultures were sent (all cultures later remained sterile). She received IV antibiotics for 14 days. Doxycycline was discontinued after seven days.

Echocardiography (ECHO) was performed to evaluate for extracardiac involvement and was normal. Joint screening for septic arthritis was also unremarkable. There was no pneumothorax, and serial monitoring demonstrated a gradual reduction of pleural collections on ICD.

The child became afebrile and clinically improved, and the ICD was removed on the sixth postoperative day. At discharge, the patient was afebrile, maintaining normal oxygen saturation on room air and active. Meropenem was discontinued once clinical improvement was sustained, and the child was continued on oral linezolid for 14 more days.

**Figure 4:**
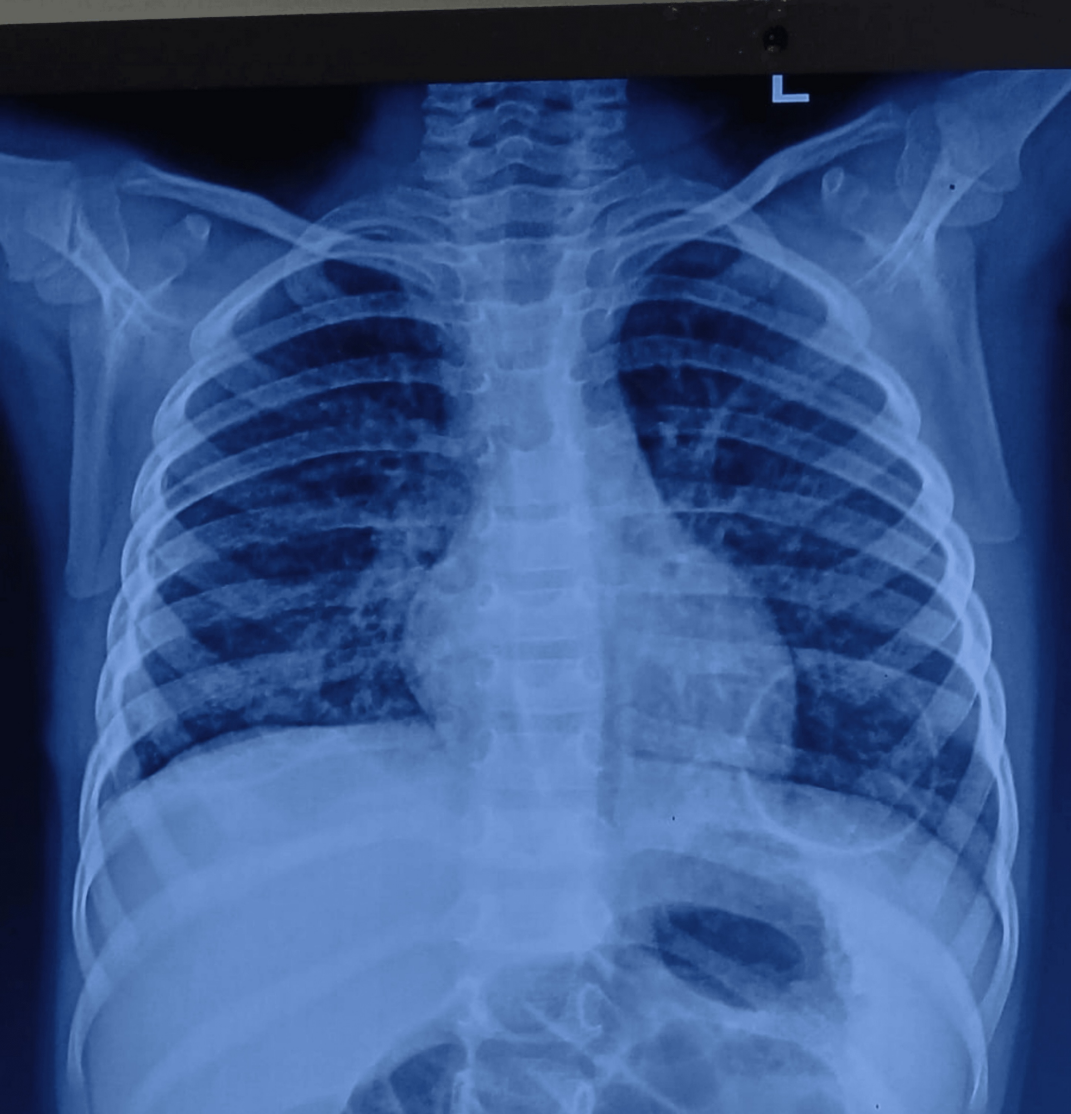
Chest radiograph obtained two months after surgery Chest radiograph obtained two months after surgery shows complete re-expansion of the right lung with significantly improved aeration bilaterally. Residual right-sided cavitary changes are still visible but markedly decreased. No pleural effusion or new consolidations are seen.

At the two-month follow-up, the chest radiograph showed normal lung aeration with complete right lung re-expansion and no residual effusion, confirming sustained recovery (Figure 4).

To evaluate for any underlying immunodeficiency, a comprehensive immunological assessment was performed at the two-month follow-up. Serum immunoglobulin levels (IgG, IgA, IgM, IgE) were within age-appropriate reference ranges, and lymphocyte subset analysis demonstrated normal distributions of T cells, B cells, and natural killer (NK) cells. Whole exome sequencing did not identify any pathogenic or likely pathogenic variants associated with primary immunodeficiency. These results collectively confirmed that the child was immunocompetent. Laboratory findings from the two-month follow-up are summarized in Table 2.

**Table 2:** Results of the two-months follow-up immunological testing Follow-up immunological testing, including immunoglobulin profile, lymphocyte subset analysis, and whole exome sequencing, demonstrated values within expected age-matched ranges and did not reveal evidence of an underlying immunodeficiency. IgG - immunoglobulin G; IgA - immunoglobulin A; IgM - immunoglobulin M; IgE - immunoglobulin E; CD - cluster of differentiation; NK cells - natural killer cells; T cell - T lymphocyte; B cell - B lymphocyte

Parameter	Result	Reference range	Unit
IgG	820	370-1560	mg/dL
IgA	58	20-150	mg/dL
IgM	96	40-160	mg/dL
IgE	22	<60	IU/mL
Total lymphocyte count	4200	2000-8000	cells/µL
CD3 T cells	2900 (69%)	55-75% (1200-4800)	cells/µL (%)
CD4 T cells	1650 (39%)	30-55% (900-2500)	cells/µL (%)
CD8 T cells	870 (21%)	15-30% (500-1500)	cells/µL (%)
CD19 B cells	620 (15%)	10-25% (200-1100)	cells/µL (%)
CD16/56 NK cells	310 (7%)	5-20% (100-600)	cells/µL (%)
Whole exome sequencing	No pathogenic or likely pathogenic variants detected; no immunodeficiency syndrome identified	-	-

## Discussion

This case illustrates necrotizing pneumonia developing as a complication of viral-bacterial co-infection in a previously healthy, immunized child. Although the introduction of pneumococcal conjugate vaccines has significantly reduced invasive pneumococcal disease, NP continues to be reported, particularly in infections involving non-vaccine serotypes or concurrent viral pathogens.

In this child, the combination of Streptococcus pneumoniae antigen positivity and co-infection with influenza, parainfluenza, and rhinovirus supports the established concept of viral-bacterial synergism. Viral pathogens compromise mucociliary function, disrupt epithelial barriers, and alter host immune responses, thereby facilitating deeper bacterial invasion and promoting parenchymal necrosis [8].

Radiologically, NP is characterized by dense consolidation, cavitary destruction, and loss of normal lung architecture. Contrast-enhanced CT remains the most sensitive modality for defining these changes and, in this case, demonstrated extensive multilobar cavitations and a loculated effusion.

Despite early initiation of broad-spectrum antibiotics, the child demonstrated persistent fever, systemic toxicity, and worsening respiratory distress, along with increasing pleural complexity on serial ultrasound. Failure to show improvement within 48-72 hours of adequate therapy is a recognized marker of complicated pneumonia and warrants reassessment for pleural space disease. Blood and pleural fluid cultures remained sterile, likely reflecting prior antibiotic exposure and the loculated nature of the empyema. In this context, ongoing sepsis in the presence of organized pleural fluid and evolving cavitation is an accepted indication for surgical intervention [9]. Necrotic lung tissue is poorly perfused, limiting antibiotic penetration and explaining the inadequate response to intravenous antibiotics alone [10]. Timely surgical management is therefore essential to halt progression and restore lung expansion. Video-assisted thoracoscopic surgery decortication was therefore undertaken and provided effective source control.

This case further highlights the diagnostic challenges inherent in culture-negative NP and underscores the importance of integrating clinical features, antigen detection, and radiologic findings when deciding on escalation of care. A multidisciplinary approach involving pediatricians, radiologists, infectious disease specialists, and surgeons is often required to guide management. Early identification of deterioration, judicious imaging, and prompt surgical intervention can substantially improve outcomes in NP [11].

To exclude an underlying predisposition, a comprehensive immunological evaluation, including immunoglobulin profile, lymphocyte subset analysis, and whole exome sequencing, was performed during convalescence. All results were within age-appropriate ranges, confirming that the child did not have an underlying immunodeficiency.

## Conclusions

Necrotizing pneumonia is an uncommon but serious complication of community-acquired pneumonia, characterized by parenchymal necrosis with liquefaction and cavitation. Viral bacterial co-infection, most often involving Streptococcus pneumoniae or Staphylococcus aureus, remains a major pathogenic mechanism and can occur even in appropriately vaccinated children. The limited response to antibiotics seen in necrotizing pneumonia is attributable to vascular thrombosis and impaired perfusion within necrotic lung tissue, which restricts antimicrobial penetration. Accordingly, early recognition, vigilant monitoring of clinical parameters and inflammatory markers, and timely surgical intervention, such as VATS decortication, are essential to prevent clinical deterioration and promote recovery.

In this case, although microbiological cultures were sterile, likely due to prior antibiotic exposure, the combination of a positive pneumococcal antigen result and characteristic CT findings strongly supported the diagnosis. As reflected in existing literature, negative cultures should not delay escalation of management when clinical, radiologic, and antigen-based evidence is compelling. Early, decisive surgical intervention was key to achieving a favorable outcome in this child.
